# Shared genetics of IgA nephropathy and cardiometabolic diseases revealed by integrative genomic analysis

**DOI:** 10.1080/0886022X.2026.2706884

**Published:** 2026-07-31

**Authors:** Lu Dai, Guanglei Chen, Yinan Yang, Yiping Yan, Qin Zhao, Na He, Yongjing Xiang, Zhengsheng Li, Yunzhi Chen

**Affiliations:** Guizhou University of Traditional Chinese Medicine, Guiyang, China

**Keywords:** IgA nephropathy, pan-vascular diseases, GWAS, genetic correlation, integrative genomics, drug repurposing

## Abstract

Immunoglobulin A nephropathy (IgAN), the most common primary glomerular disease and a leading cause of end-stage kidney disease (ESKD), is associated with cardiovascular and metabolic abnormalities. To explore these genetic overlaps, we integrated large-scale GWAS data from IgAN, 21 pan-vascular diseases (PVDs), 13 metabolic traits, 11 immune cell phenotypes, and established IgAN risk factors. Cross-trait analyses (LDSC, GNOVA, SUPERGNOVA) revealed significant genetic correlations between IgAN and multiple PVDs, including coronary artery disease, heart failure, stroke, aortic aneurysm, and varicose veins. Multi-omics prioritization (TWAS, SMR, MAGMA, and fastBAT) identified shared variants and candidate genes associated with immune regulation, lipid transport, and cardiovascular function. Gene expression profiling demonstrated enrichment of these genes in endothelial cells, macrophages, spleen, lung, and blood. Drug repurposing analysis based on gene–drug matching scores (>0.5) identified promising therapeutic candidates, including immunosuppressants (prednisolone, cyclosporine, azathioprine), lipid-lowering agents (pitavastatin, fenofibrate), and the antiplatelet drug aspirin. These findings offer novel opportunities for precision medicine and drug repurposing in IgAN patients with cardiometabolic disorders.

## LAY (PLAIN LANGUAGE) SUMMARY

Immunoglobulin A nephropathy (IgAN) is the most common form of primary kidney disease worldwide and a leading cause of kidney failure. Increasing evidence suggests that IgAN is not limited to the kidneys but is also associated with cardiovascular and metabolic diseases, including coronary artery disease, stroke, heart failure, and abnormal lipid metabolism. However, the genetic mechanisms underlying these associations remain poorly understood. In this study, we analyzed large-scale genetic datasets from IgAN, vascular diseases, metabolic traits, and immune-related phenotypes to investigate their shared biological basis. We found that IgAN shares significant genetic overlap with several cardiovascular diseases and lipid-related traits. Multiple shared genes were identified that are involved in immune regulation, inflammation, endothelial dysfunction, and lipid metabolism. Importantly, several existing drugs, including statins, aspirin, and immunosuppressive agents, were predicted to have potential therapeutic value for IgAN patients with cardiovascular or metabolic complications. These findings improve our understanding of the systemic nature of IgAN and may help support the development of precision medicine strategies and drug repurposing approaches for patients with IgAN.

## KEY MESSAGES/IMPACT STATEMENT

**What is known:** Immunoglobulin A nephropathy (IgAN) is increasingly recognized as a systemic immune-mediated disorder associated with cardiovascular and metabolic abnormalities; however, the underlying shared genetic mechanisms remain incompletely elucidated.

**What this study adds:** By integrating multi-omics approaches with large-scale GWAS datasets, this study identified significant genetic correlations, shared susceptibility loci, and convergent biological pathways linking IgAN with pan-vascular diseases, lipid metabolic traits, and immune phenotypes.

**Potential impact:** These findings further advance the understanding of the shared genetic architecture between IgAN and cardiovascular-metabolic disorders, thereby providing novel insights into precision medicine and supporting mechanism-based drug repurposing strategies for IgAN patients with vascular and metabolic comorbidities.

## GRAPHICAL ABSTRACT

**Figure:**
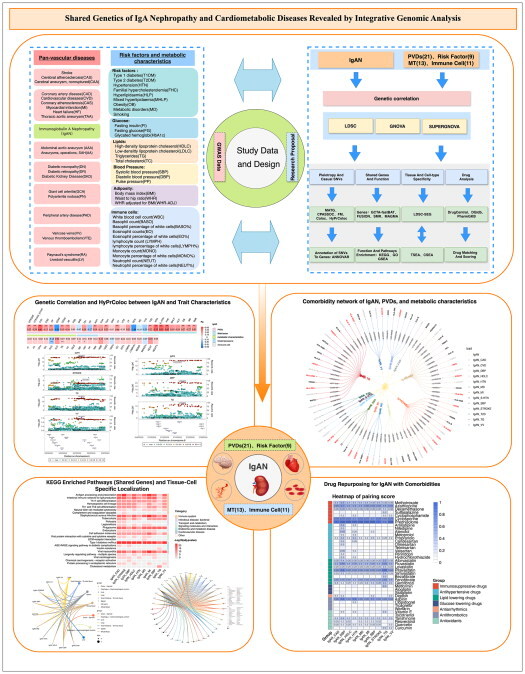

## 1. Introduction

Immunoglobulin A nephropathy (IgAN) is the most prevalent primary glomerular disease worldwide, characterized by a progressive decline in renal function, which significantly increases the risk of end-stage kidney disease (ESKD) and all-cause mortality [1]. Despite substantial advances in understanding the molecular mechanisms underlying IgAN in recent years, its complex etiology, diverse regulatory mechanisms, and considerable interpatient variability continue to pose significant challenges. Consequently, current therapeutic strategies have yet to achieve substantial breakthroughs. Even with the implementation of optimal standardized supportive treatments, approximately 30%–40% of patients progress to ESKD within 20 years, presenting a considerable challenge for clinical management [2].

Accumulating clinical evidence indicates that patients with IgAN are at a significantly increased risk of developing cardiometabolic complications compared with the general population. Epidemiological studies have consistently demonstrated a higher prevalence of hypertension, dyslipidemia, insulin resistance, obesity, metabolic disturbances, and cardiovascular events among patients with IgAN [3–6]. Notably, cardiovascular complications have emerged as one of the leading causes of mortality in this population[7]. Collectively, these findings suggest that, in addition to localized renal immune injury, IgAN may also be associated with systemic pathological processes involving cardiorenal metabolic dysregulation and vascular injury. The pathogenesis of IgAN remains incompletely understood. Its complex pathophysiological processes are thought to result from the interplay of multiple factors, including immune dysregulation, genetic susceptibility, environmental exposures, and metabolic imbalances [3]. In recent years, growing attention has been directed toward the potential role of lipid metabolism abnormalities in the progression of IgAN. Existing studies have demonstrated that abnormal lipid deposition within the renal parenchyma can induce the formation of a localized inflammatory microenvironment, which subsequently promotes renal fibrosis [8]. Some studies suggest that there may be shared pathophysiological mechanisms between glomerulosclerosis and atherosclerosis [6]. Notably, pan-vascular diseases (PVDs), defined by atherosclerosis as a central pathological feature, such as coronary artery disease and cerebrovascular diseases [9], share typical risk factors – including smoking, lipid metabolism disorders, and hypertension – that are also prevalent among IgAN patients [10–13]. This clinical phenomenon suggests that IgAN may share common metabolic abnormalities with PVDs. Given this background, the present study aims to systematically investigate the potential shared genetic susceptibility and molecular associations between IgAN and various PVDs, with a particular focus on lipid metabolism disorders. By examining these interrelationships from genetic and multi-omics perspectives, this study seeks to provide new theoretical insights and identify candidate targets for developing intervention strategies aimed at common metabolic pathways involved in these conditions.

Similar to other immune-mediated complex diseases, IgAN is characterized as a typical polygenic disorder, with its phenotype influenced by both genetic and environmental factors. Genetic factors play a pivotal role in the pathogenesis of IgAN, as evidenced by several multicenter studies that have reported familial IgAN cases. For instance, epidemiological data from specific regions, such as Northern Italy, Southern France, and Eastern Kentucky in the United States, suggest that familial aggregation accounts for approximately 10%–15% of the total cases in these areas [14–17]. However, high heritability is a phenomenon more commonly observed in cardiovascular and metabolic diseases [18], further underscoring the significant value of genetic research in elucidating the mechanisms of complex diseases [19]. In recent years, with the expansion of genome-wide association studies (GWAS), researchers have successfully identified a substantial number of single nucleotide polymorphisms (SNPs) associated with susceptibility to various complex diseases, including both IgAN and PVDs, using large-scale case-control studies or population cohorts [20, 21]. These findings not only deepen our understanding of the molecular underpinnings of these diseases but also provide theoretical support for the development of risk prediction models based on genetic markers and the formulation of precision medicine strategies.

Consequently, this study aims to systematically identify and validate shared genetic susceptibility loci and potential causal variants between IgAN and various PVDs by integrating large-scale, published summary data from GWAS. The objectives of the study include the identification of shared single nucleotide variants (SNVs) and associated genes, as well as the analysis of significantly enriched biological pathways, tissue-specific, and cell type-specific expression patterns. Notably, this research provides theoretical support for the prioritization of drug development targeting shared genetic mechanisms in IgAN, and offers novel insights for optimizing therapeutic strategies for IgAN in conjunction with metabolic disorders in PVDs.

## 2. Methods

### 2.1. Study design and GWAS summary data

**Figure 1.**
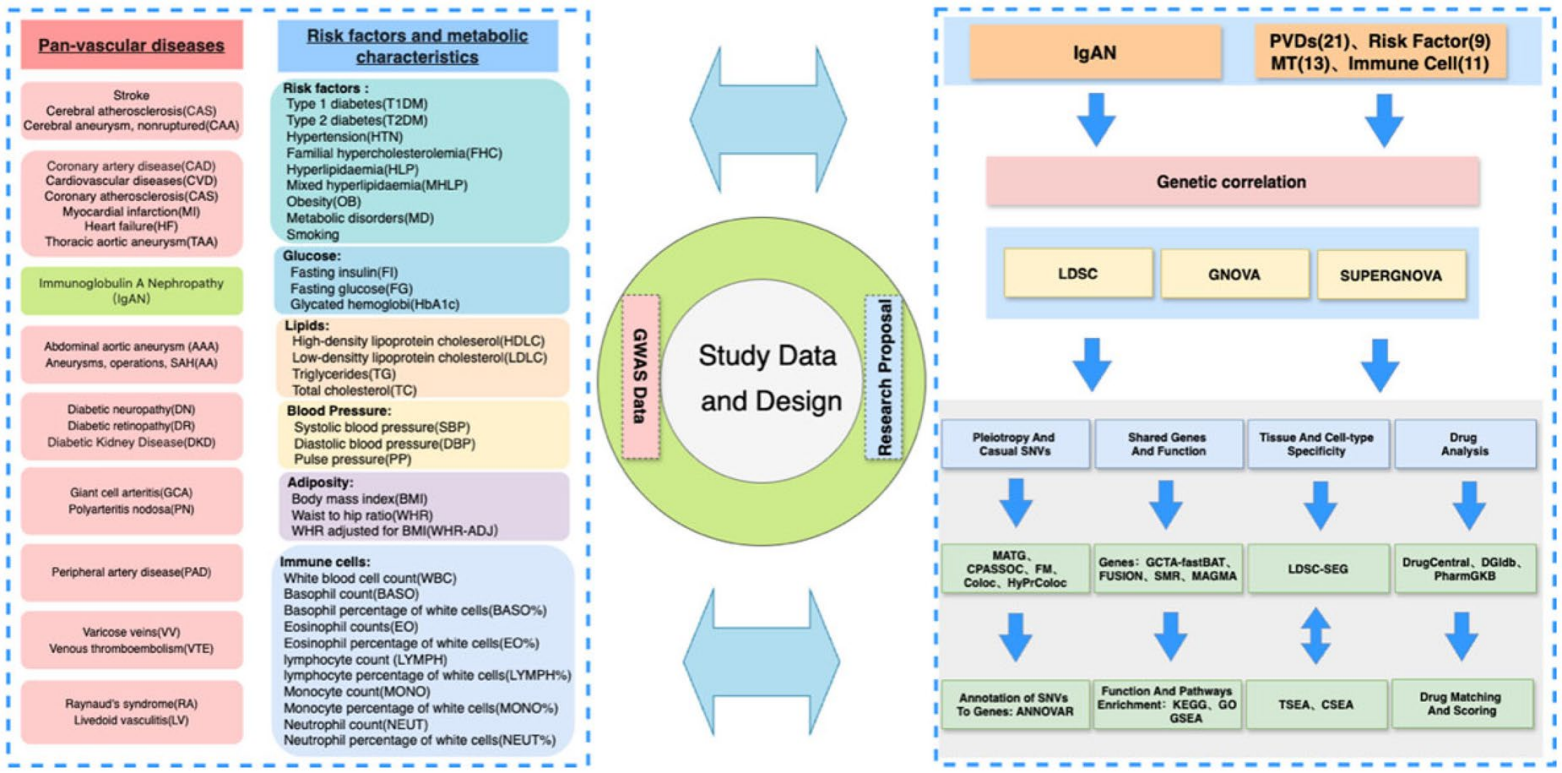
Research workflow diagram. Note: Study Data: The diseases and trait characteristics in the study include IgAN and 21 types of PVDs, 8 IgAN-related risk factors, 13 metabolic traits, and 11 immune cell characteristics. This workflow includes calculating genome-wide genetic correlations between diseases and traits using LDSC, GNOVA, and SUPERGNOVA, identifying shared causal variants, genes, and pathways, discovering the tissues and cells most significantly affected by shared signals, as well as prioritizing drugs for the treatment of IgAN comorbidities. Abbreviations: IgAN: Immunoglobulin A Nephropathy, PVDs: Pan-Vascular Diseases, MT: Metabolic Traits, LDSC: Linkage Disequilibrium Score Regression, GNOVA: Genetic Covariance Analyzer, MTAG: Multi-Trait Meta-Analysis, FM: Fine-Mapping, TSEA: Tissue-Specific Expression Analysis, CSEA: Cell-Type Specific Enrichment Analysis.

All GWAS summary statistics were obtained from the GWAS Catalog (https://www.ebi.ac.uk/gwas/home) and FinnGen (https://www.finngen.fi/en). Summary-level data on immune cell traits were retrieved from the Blood Cell Consortium (BCX) [22]. The types of data analyzed and the overall study design are illustrated in Figure 1. Detailed information regarding the datasets and their respective sources is provided in Supplementary Table 1.

### 2.2. Genetic correlation analysis

In this study, we systematically assessed the genetic correlations between IgAN and PVDs, metabolic traits, immune cell characteristics, and various risk factor-related phenotypes by leveraging publicly available GWAS summary statistics. Linkage Disequilibrium Score Regression (LDSC) [23] was employed to quantify the extent to which linkage disequilibrium (LD) scores contribute independently to phenotypic variation, thereby elucidating the underlying polygenic architecture of complex traits. During the analytical process, rigorous adjustments were made to account for potential confounding effects arising from sample size heterogeneity, measurement errors, and population stratification. This approach enabled the construction of a robust model for estimating genetic correlation.

To ensure statistical reliability, we adopted a dual-significance threshold: a heritability estimate (*h*^2^) with a *p*-value < 0.05 was required, alongside a genetic correlation coefficient (*r*_g_) *p*-value also < 0.05. To further minimize false-positive discoveries due to multiple comparisons, all *p*-values were adjusted using the False Discovery Rate (FDR) method. Only genetic correlations with both *h*^2^ and *r*_g_ FDR-adjusted *p*-values (*P*_FDR_) < 0.05 were considered statistically significant. LDSC analysis was conducted using the publicly available software package (https://github.com/bulik/ldsc). The genetic *r*_g_ ranges from −1 to 1, with an *r*_g_ of 1 indicating a complete positive genetic correlation between traits, and an *r*_g_ of −1 representing a complete negative (inverse) genetic correlation.

To further elucidate the shared and distinct genetic architecture between IgAN and PVDs, we employed the GeNetic cOVariance Analyzer (GNOVA) algorithm [24] within the framework of Annotated Stratified Genetic Covariance Estimation. The GNOVA software is publicly available at https://github.com/xtonyjiang/GNOVA. By integrating functional genomic annotation data across stratified layers, GNOVA enables more refined quantification of heritability for IgAN and related traits, while also delineating the specific features of genetic correlation among different phenotypes. Notably, the results obtained using the GNOVA method demonstrated a high degree of concordance with those derived from LDSC analysis, both in the directionality and statistical significance of the estimated genetic correlations between IgAN and PVDs (*P*_FDR_ < 0.05 for both methods). This methodological consistency further reinforces the robustness and credibility of our findings across complementary analytical approaches.

To achieve a more granular characterization of local genetic correlations within specific genomic regions, we employed the SUPERGNOVA (SUPER GeNetic cOVariance Analyzer) algorithm [25], available at https://github.com/qlu-lab/SUPERGNOVA. By jointly modeling the GWAS summary statistics for IgAN and PVDs, SUPERGNOVA enables precise estimation of region-specific local genetic correlations. A major strength of this method lies in its capacity to effectively correct for estimation biases arising from local LD structures and sample overlap between studies. Statistical inference was conducted using a two-tiered significance threshold. Genomic loci with *P*_FDR_ < 0.05 were considered to exhibit statistically significant local genetic correlations. In contrast, regions with uncorrected *p*-value < 0.05 but failing to meet the FDR threshold were classified as suggestive signals.

### 2.3. Multi-Trait meta-analysis

To systematically investigate the shared genetic mechanisms between different traits, particularly between IgAN and PVDs as well as other related phenotypes, we employed a multi-trait meta-analysis (MTAG) framework. This approach integrates summary data from multiple GWAS studies to enhance the robustness of the findings. MTAG utilizes a generalized inverse-variance weighting algorithm to conduct joint analyses across traits. The key advantages of this method are outlined as follows [26]: (i) it effectively corrects for potential bias in parameter estimation that may arise from sample overlap between different GWAS studies; (ii) it constructs a joint statistical model based on the variance-covariance matrix of SNVs effect sizes; (iii) as a consistent estimator, MTAG improves statistical power – especially for traits with smaller sample sizes – while simultaneously reducing the mean squared error (MSE) across the entire genome, and effectively mitigates the FDR inflation issues that can arise when analyzing highly correlated traits. (MTAG: https://github.com/JonJala/mtag).

Given the assumptions underlying the MTAG method, such as the homogeneity of SNP heritability across traits and the constancy of genetic covariance, there are certain limitations in its practical application. Consequently, in this study, we employed cross-phenotype association analysis (CPASSOC) as a sensitivity analysis to validate the robustness of the MTAG results [27] (CPASSOC: https://hal.case.edu/∼xxz10/zhu-web/). CPASSOC integrates GWAS data from multiple traits through a Bayesian mixture model, enabling the detection of shared genetic variations across traits while effectively controlling for population stratification and potential cryptic relatedness. CPASSOC incorporates a dual testing framework: the first, SHom (Homogeneous Effect Model), utilizes a fixed-effects model designed to maximize the weighted sum of trait-specific genetic effects; however, this model is sensitive to heterogeneity across studies. The second, SHet (Heterogeneous Effect Model), employs a random-effects model that accounts for variability in effect sizes introduced by factors such as differences in study design, environmental exposures, and phenotypic heterogeneity. In this study, we performed a comparative evaluation of the SHom and SHet models based on the Akaike Information Criterion (AIC) and ultimately selected the SHet model as the framework for the primary inferential analysis.

Following the cross-trait meta-analysis, the present study employed the PLINK software (version 1.9; PLINK: https://www.cog-genomics.org/plink/1.9) to perform independence screening (clumping) of the identified SNVs. The filtering parameters were set as follows: the significance threshold for index SNPs (–clump-p1) was set to 5 × 10^−8^, the secondary SNP significance threshold (–clump-p2) to 1 × 10^−5^, the linkage disequilibrium r^2^ threshold (–clump-r^2^) to 0.1, and the physical distance window (–clump-kb) to 1000 kb. Genetic variants that simultaneously met the following two criteria were defined as loci with significant pleiotropy: first, they achieved genome-wide significance (*p* < 5 × 10^−8^) in the original univariate GWAS analysis; and second, they reached genome-wide significance in both MTAG and CPASSOC meta-analysis results (i.e. PMTAG < 5 × 10^−8^ and PCPASSOC < 5 × 10^−8^). Subsequently, the identified pleiotropic variants were subjected to comprehensive functional annotation using the ANNOVAR software [28]. To visually represent the complex associations between these variants and various phenotypes, we constructed a multi-level SNVs-phenotype association network using the ggraph package in R. The network topology was optimized using the Fruchterman-Reingold algorithm, and the degree centrality of each node in the network was employed to quantify the pleiotropic strength of the corresponding SNVs or its centrality within the association network.

### 2.4. Fine-Mapping credible set analysis

To further dissect the potential causal variants located within loci identified by cross-trait genetic association analyses (e.g. MTAG or CPASSOC), we employed the FM-summary method, a Bayesian fine-mapping approach [29]. This method is based on a variational inference algorithm and was implemented using the FM-summary toolkit (https://github.com/hailianghuang/FM-summary). By constructing complex Bayesian models, FM-summary effectively distinguishes true causal signals from numerous non-causal variants that are in high LD with the actual causal variants. The analytical workflow was as follows. First, for shared SNPs significantly associated with multiple traits as identified by the cross-trait meta-analysis, we extracted all genetic variants located within *a* ± 500 kb genomic region centered on each index SNP. These variants served as input for the fine-mapping procedure. Second, a flat (uninformative) prior distribution was assigned to each variant during the FM-summary analysis, and the posterior inclusion probability (PIP) was calculated. The PIP quantifies the probability that a given variant is one of the causal variants within the region. Finally, all variants were ranked by their PIP values and accumulated iteratively to construct a 99% credible set – defined as the smallest subset of variants whose combined PIP accounts for at least 99% of the posterior probability of the observed association signal. This method demonstrates robust statistical performance by simultaneously incorporating effect size estimates from genome-wide association studies (β coefficients and their standard errors) alongside detailed local LD structure within the genomic region of interest.

### 2.5. Colocalization analysis

To investigate whether IgAN and specific clinical traits – such as PVDs – share common causal genetic variants, we employed a two-stage Bayesian statistical framework to assess the presence of genetic colocalization. In the first stage, we performed pairwise colocalization analyses using the *coloc* R package (https://github.com/chr1swallace/coloc) [30]. Specifically, for each indicative SNVs previously identified through cross-trait analyses (e.g. MTAG or CPASSOC) or fine-mapping approaches (e.g. FM-summary), we extracted all genetic variants located within *a* ± 500 kb region flanking the target SNVs. Corresponding GWAS summary statistics for both IgAN and the clinical trait of interest were retrieved for these regions.The *coloc* method evaluates five competing hypotheses by computing their posterior probabilities, with particular emphasis on the posterior probability of hypothesis 4 (P(H4)), which posits that the two traits share a single causal variant within the specified locus [31]. A P(H4) value exceeding 0.6 – or a more stringent threshold, depending on the context of the study – was considered indicative of significant evidence for colocalization.

In the second stage of colocalization analysis, to further investigate whether more than two traits share causal variants within specific genomic regions, we conducted multi-trait colocalization analysis using the HyPrColoc tool (version 1.0.0) within the R environment (version 4.2.3) (https://github.com/cnfoley/hyprcoloc). HyPrColoc implements a Bayesian hierarchical clustering algorithm to estimate the joint posterior probability (PP) that a set of traits colocalize at one or more causal variants within a given locus and simultaneously identifies clusters of traits that share these variants. This integrative analytical framework leverages both GWAS-derived effect size estimates across traits and the local LD structure to enhance the resolution of causal inference. Consequently, it improves the ability to distinguish true pleiotropic loci – where a single variant influences multiple traits – from spurious colocalization signals that may arise due to tight LD among nearby variants. Hence, this method offers improved accuracy in identifying biologically meaningful shared genetic architecture across complex traits.

### 2.6. Multidimensional gene-level association analysis

To systematically elucidate the shared genetic architecture between IgAN and clinical traits, a multidimensional gene-level analytical strategy was employed. Specifically, the following four complementary approaches were utilized: ① Transcriptome-Wide Association Study (TWAS): TWAS was performed using the FUSION software package, which integrates GWAS summary statistics with pre-constructed gene expression prediction models based on expression quantitative trait loci (eQTL) data from 49 human tissues in the GTEx v8 project (http://gusevlab.org/projects/fusion/). This approach evaluates whether the genetically predicted expression levels of genes are significantly associated with the target traits [32]. ② Summary-data-based Mendelian Randomization (SMR): SMR analysis was conducted to assess whether shared genetic variants influence both gene expression and clinical phenotypes. This method combines GWAS summary statistics with eQTL data derived from the eQTLGen consortium (whole blood) and GTEx v8 (49 tissues) (https://cnsgenomics.com/software/smr/#Overview). The heterogeneity in dependent instruments (HEIDI) test (*P*_HEIDI > 0.05) was applied to distinguish true pleiotropic associations from spurious signals arising due to LD [33,34]. ③ Multi-marker Analysis of GenoMic Annotation (MAGMA): Gene-level association analysis was conducted using MAGMA (https://ctg.cncr.nl/software/magma), which maps SNPs to genes based on their physical location (e.g. within ±20 kb of gene boundaries) and aggregates SNP-level association signals through a multiple regression framework that accounts for LD structure using the 1000 Genomes Project European reference panel [35]. ④ GCTA-fastBAT: The fastBAT module in the GCTA (Genome-wide Complex Trait Analysis) software suite (https://yanglab.westlake.edu.cn/software/gcta/#fastBAT) was employed to assess the joint effects of multiple variants within gene regions. This method, based on the Burden/SKAT test framework, uses an approximate chi-squared distribution of GWAS summary statistics and incorporates the same LD reference panel as MAGMA for efficient evaluation of gene-level associations [36].

For all four approaches, gene–trait association *p*-values were adjusted using FDR correction. Genes that reached statistical significance (*P*_FDR_ < 0.05) in at least three of the four methods were defined as high-confidence shared genes.

### 2.7. Multidimensional enrichment and expression analysis

To gain deeper insight into the potential biological functions and mechanistic roles of the previously identified high-confidence shared genes – i.e. genes exhibiting strong association signals with both IgAN and key clinical traits such as PVDs – we performed a series of multidimensional enrichment and expression profiling analyses. These analyses were designed to elucidate the cellular, tissue-specific, and pathway-level enrichment patterns of the shared genes, thereby providing a more comprehensive understanding of their biological relevance within the context of IgAN pathogenesis and associated phenotypes.

1. **Cell-Type Specific Enrichment Analysis (CSEA):** To evaluate whether the identified shared genes exhibit cell-type-specific expression or enrichment, we performed cell-type specific enrichment analysis using the WebCSEA online platform (https://bioinfo.uth.edu/webcsea). This tool integrates transcriptomic data from 53 human tissues derived from the Genotype-Tissue Expression (GTEx v8) project [37], along with epigenomic features (e.g., chromatin accessibility) from the CATLAS single-cell atlas of kidney cell types [38]. Through this integrative approach, WebCSEA enables the systematic assessment of gene set specificity across a broad range of human cell types, thereby facilitating the identification of cell populations in which the input gene set is significantly overrepresented.
2. **Tissue-Specific Expression Analysis (TSEA):** Tissue-specific expression analysis was conducted using the TSEA tool (available at http://doughertylab.wustl.edu/tsea) to determine whether the identified shared genes exhibit significant expression enrichment in particular human tissues. The analysis utilized transcriptomic data from the GTEx project, encompassing expression profiles across 45 major tissues, derived from 1,839 RNA-seq samples collected from 189 postmortem donors [39]. A hypergeometric test was employed to statistically evaluate the degree of enrichment of the shared gene set within each tissue, thereby identifying tissues in which these genes are preferentially expressed.
3. **Pathway and Functional Enrichment Analysis:** To further elucidate the biological processes and signaling pathways associated with the identified shared genes, a comprehensive pathway enrichment analysis was performed using the *clusterProfiler* package (version 4.0) in R [40]. The input gene list consisted of the high-confidence shared genes identified in the previous analyses. To ensure analytical rigor and minimize bias, the background gene set (universe) was defined as all genes tested in at least one of the aforementioned gene-level analytical methods. The enrichment analysis encompassed the following components: a. **Gene Ontology (GO) Functional Annotation:** This included assessment of gene enrichment across three GO categories – Biological Process (BP), Molecular Function (MF), and Cellular Component (CC). b. **Kyoto Encyclopedia of Genes and Genomes (KEGG) Pathway Analysis:** This analysis was conducted to identify overrepresentation of shared genes within known metabolic and signal transduction pathways.

All enrichment analyses were subjected to multiple testing correction using the Benjamini-Hochberg procedure to control the FDR. Enrichment terms or pathways with FDR-adjusted *p*-values less than 0.05 (*P*_FDR_ < 0.05) were considered statistically significant.

Finally, to visually illustrate the complex interaction network between the identified shared genes and significantly enriched biological pathways, we employed Cytoscape software (version 3.8.2) to construct and optimize the gene-pathway interaction network. This network diagram effectively delineates the specific genes involved in key pathways and highlights the potential interconnections between different pathways.

### 2.8. Shared gene-drug target analysis

Based on the IgAN-associated genes identified by four independent gene analysis methods, a total of 56 core disease-related genes were selected for this study. To establish a gene-drug association network, we adopted a multi-dimensional biological pathway analysis approach. Initially, we conducted pathological pathway enrichment analysis on the shared gene set using the clusterProfiler tool to elucidate the molecular mechanisms underlying the clinical phenotypes of IgAN. The drug screening process was conducted in three phases: (1) Integration of three authoritative databases – DrugCentral [41], DGIdb [42], and PharmGKB [43] – was employed to search for compounds targeting the identified genes, resulting in an initial screening of 204 candidate drugs; (2) Based on clinical treatment guidelines for PVDs and clinical trial data for IgAN, 40 drugs were selected according to one of the following criteria: (i) compounds approved for the treatment of PVDs, or (ii) clinically advanced drugs with documented therapeutic potential for IgAN; (3) A paired scoring algorithm [44] was applied to quantify the functional correlation between pathological and pharmacological pathways, thereby constructing a multi-omics association map for IgAN phenotypes and drugs.

## 3. Results

### 3.1. Genome-Wide Association study dataset

This study collected summary statistics from GWAS for IgAN, 21 PVDs, 8 IgAN-associated risk factors, 13 metabolic traits (MT), and 11 immune cell characteristics (Figure 1). These traits encompass a broad range of biological domains, including cardiovascular, arterial, venous, vascular circulation, immune responses, and lipid-glucose metabolism. Based on these disease and trait-related data, we designed a comprehensive research workflow (Figure 1) aimed at systematically dissecting the shared genetic architecture between IgAN and PVDs, along with their risk factors, metabolic profiles, and immune cell features. This includes common SNVs, associated genes, pathways, tissues, and cell types.

### 3.2. Genetic regression analysis

**Figure 2.**
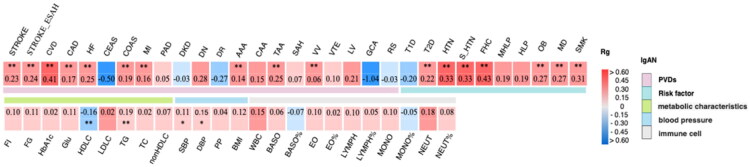
Heatmap of LDSC analysis results. Note: The heatmap displays the genetic correlation coefficients (*r*_g_) calculated by LDSC between IgAN and PVDs as well as metabolic traits. The color scale represents the strength of the correlation, with *r*_g_ values shown beside the heatmap. * indicates statistical significance: *: *P* < 0.05; **: *P* < 0.001.

LDSC-based GWAS data analysis revealed significant genetic correlations between IgAN and 21 clinical traits, including 10 PVDs, 7 IgAN-related risk factors, and 4 metabolic traits (Figure 2). Among these, cardiovascular diseases (CVD) exhibited the strongest genetic association with IgAN, followed by thoracic aortic aneurysm (TAA), heart failure (HF), stroke, coronary atherosclerosis (COAS), coronary artery disease (CAD), myocardial infarction (MI), abdominal aortic aneurysm (AAA), and varicose veins (VV). In comparison to disease traits, risk factors showed a stronger genetic correlation with IgAN, with hypertension (HTN), type 2 diabetes mellitus (T2DM), familial hypercholesterolemia (FHC), obesity (OB), secondary hypertension (S-HTN), metabolic disorder (MD), and smoking ranking in descending order. Metabolic traits demonstrated a relatively weaker genetic correlation with IgAN, with triglycerides (TG) showing a positive association and HDL cholesterol (HDL-C) a negative association with IgAN. Notably, glucose levels and immune cell traits did not meet the statistical significance threshold (*P*_FDR_ > 0.05) and were therefore excluded from subsequent analyses. Detailed LDSC results for all traits are provided in Supplementary Data 2 and Supplementary Data 3.

Subsequently, we employed GNOVA analysis, a method that demonstrates greater robustness under moderate genetic correlation, to assess the genetic covariance between IgAN and clinical traits. Consistent with the trends observed in LDSC results, the traits that were found to be significantly associated in the LDSC analysis also passed the FDR correction threshold in the GNOVA analysis (Table 1). The complete GNOVA analysis results for all traits are summarized in Supplementary Data 4.

**Table 1.** **Genetic regression analysis**.

	LDSC			GNOVA					
	*r_g_*	*P*	*P*_FDR_	*r_g_* uncorrected	*r_g_* corrected	*P* uncorrected	*P* corrected	*P*_FDR_ uncorrected	*P*_FDR_ corrected
STROKE	0.23	5.29E-04	2.69E-03	0.20	0.18	7.61E-03	1.67E-02	1.69E-02	3.27E-02
STROKE_ESAH	0.24	1.40E-03	5.90E-03	0.20	0.18	2.15E-03	3.49E-03	7.32E-03	8.48E-03
CVD	0.41	3.08E-21	1.72E-19	0.30	0.35	9.39E-10	1.51E-10	9.58E-09	1.10E-09
CAD	0.18	4.20E-06	4.70E-05	0.20	0.15	1.52E-10	2.35E-07	1.94E-09	1.09E-06
HF	0.25	4.13E-04	2.31E-03	0.16	0.15	1.84E-02	2.35E-02	3.23E-02	4.14E-02
COAS	0.19	8.64E-05	8.06E-04	0.13	0.12	6.20E-04	9.52E-04	3.16E-03	2.55E-03
MI	0.16	5.20E-03	1.71E-02	0.08	0.09	1.16E-01	8.34E-02	1.68E-01	1.25E-01
AAA	0.18	1.56E-02	4.17E-02	0.14	0.11	2.35E-03	1.57E-02	7.50E-03	3.20E-02
TAA	0.26	6.32E-04	2.95E-03	0.15	0.21	9.18E-04	1.38E-05	3.85E-03	5.04E-05
VV	0.17	9.50E-03	2.80E-02	0.11	0.19	1.86E-03	1.95E-10	6.79E-03	1.24E-09
T2D	0.22	1.30E-07	1.81E-06	0.18	0.20	1.04E-10	7.69E-12	1.77E-09	1.31E-10
HTN	0.33	2.35E-16	6.57E-15	0.26	0.29	1.22E-11	2.93E-12	3.12E-10	7.47E-11
S_HTN	0.33	4.14E-03	1.45E-02	0.25	0.25	8.67E-03	7.45E-03	1.84E-02	1.58E-02
FHC	0.44	1.47E-03	5.90E-03	0.20	0.37	5.31E-04	3.01E-11	3.01E-03	3.07E-10
OB	0.27	1.62E-12	3.03E-11	0.24	0.27	1.74E-07	4.23E-08	1.27E-06	2.16E-07
MD	0.28	1.24E-04	9.93E-04	0.20	0.17	2.71E-03	7.28E-03	8.12E-03	1.58E-02
SMK	0.32	1.77E-03	6.61E-03	0.26	0.27	7.14E-04	5.76E-04	3.31E-03	1.63E-03
HDLC	−0.16	2.01E-04	1.25E-03	−0.17	−0.12	3.55E-03	2.16E-02	9.54E-03	3.94E-02
TG	0.19	1.54E-04	1.08E-03	0.19	0.16	6.67E-09	1.08E-06	5.67E-08	4.59E-06
SBP	0.11	7.81E-03	2.43E-02	0.10	0.10	9.81E-04	1.81E-03	3.85E-03	4.61E-03
DBP	0.15	1.34E-02	3.75E-02	0.16	0.19	4.30E-03	2.14E-04	1.10E-02	6.43E-04

Note: LDSC: Linkage Disequilibrium Score Regression; GNOVA; Genetic Network-based Optimization for Variance Analysis; CVD: Cardiovascular Disease; CAD: Coronary Artery Disease; HF: Heart Failure; COAS: Coronary Atherosclerosis; MI: Myocardial Infarction; AAA: Abdominal Aortic Aneurysm; TAA: Thoracic Aortic Aneurysm; VV: Varicose Veins; T2D: Type 2 Diabetes; HTN: Hypertension; S_HTN: Secondary Hypertension; FHC: Familial Hypercholesterolemia; OB: Obesity; MD: Metabolic Disorder; SMK: Smoking; HDLC: High-Density Lipoprotein Cholesterol; TG: Triglycerides; SBP: Systolic Blood Pressure; DBP: Diastolic Blood Pressure; *r_g_*: Genetic correlation; *P*_fdr_: False Discovery Rate-corrected *p*-value.

Furthermore, we utilized SUPERGNOVA to partition the entire genome into LD-independent regions. The results revealed that the CAD-IgAN associated regions were the most abundant, with five significantly associated regions and 77 suggestive regions identified. This was followed by CVD, with three significant regions and 73 suggestive regions. Among other PVDs, AS was found to have one significant region and 59 suggestive regions, while TAA, HF, stroke, MI, and AAA only displayed suggestive associations. Consistent with the LDSC findings, the risk factor phenotypes exhibited stronger local genetic correlations with IgAN, with the following order of association strength: HTN, T2DM, FHC, OB, S-HTN, MD, and smoking. However, lipid metabolism-related phenotypes (HTN, FHC, MD) showed both greater genetic correlation strength and broader regional distribution, supporting their pivotal role in the comorbidity of IgAN and vascular disease. Notably, among the physiological traits, TG was associated with IgAN in four significant regions and 95 suggestive regions, while HDL-C was associated with IgAN in five significant regions and 75 suggestive regions. These findings provide genomic evidence for the involvement of lipid metabolism pathways in the molecular mechanisms underlying IgAN-related vascular complications (Supplementary Data 5).

### 3.3. Cross-Trait loci and causal variants

**Figure 3.**
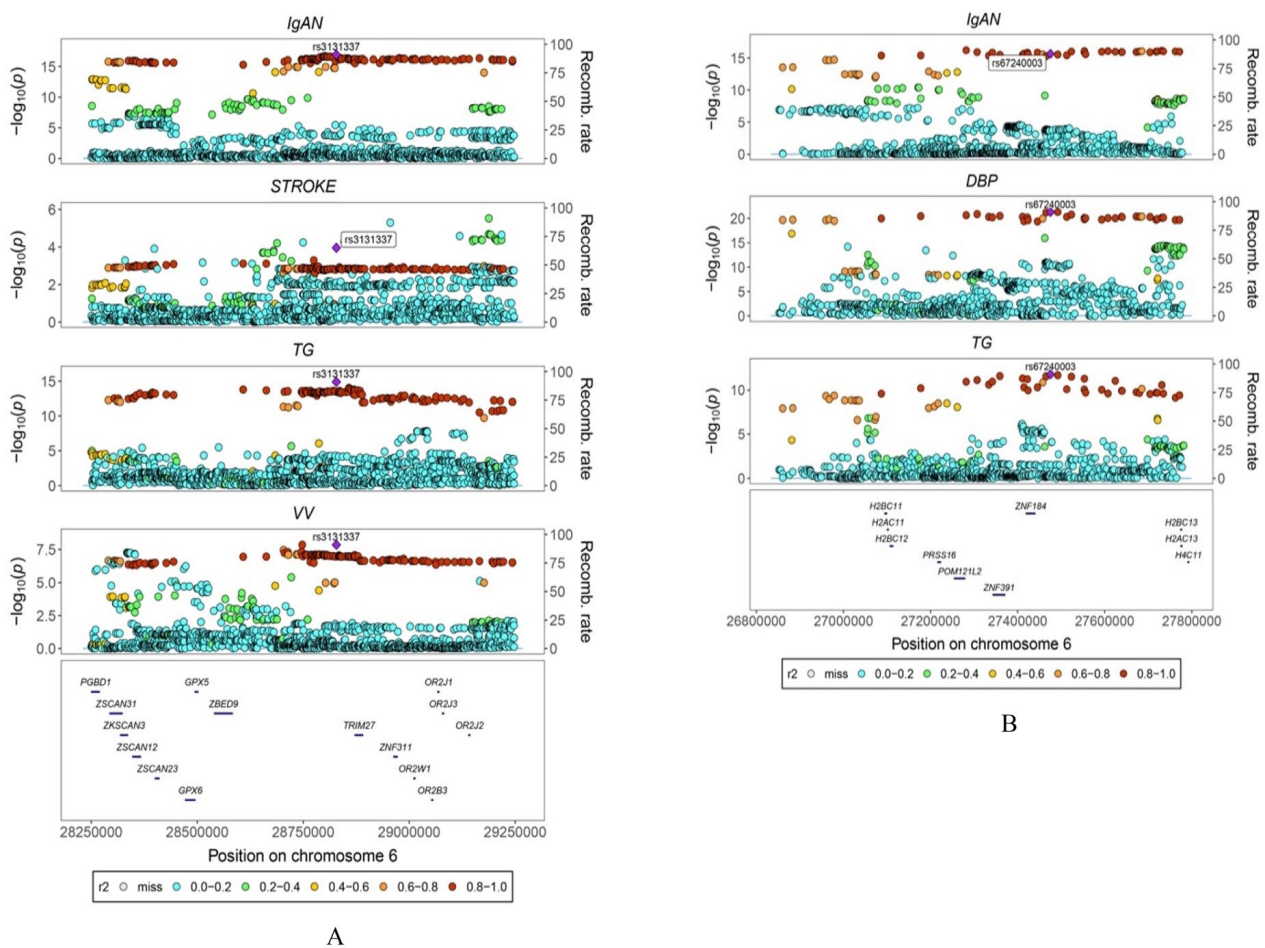
Pleiotropic colocalization analysis. **Note:** Two LocusZoom plots of pathogenic variants shared between IgAN and other traits. These variants also represent the lead single nucleotide variants in the studied regions. *P*-values are derived from the original genome-wide association studies.

By integrating cross-phenotype meta-analysis using both MTAG and CPASSOC methodologies, a total of 64 shared SNVs across 21 trait pairs were identified (Supplementary Data 6). At the disease association level, IgAN and CAD exhibited the greatest number of shared SNVs (*N* = 9). Notably, compared with disease phenotypes, the genetic overlap between IgAN and physiological traits was more pronounced. Specifically, 29 SNVs were shared with TG levels, 24 with DBP, and 17 with HDL-C. Among risk factors, IgAN shared 10 SNVs with HTN. To identify potential shared causal variants, fine-mapping of the target SNVs was performed using the FM-summary method (99% credible set), followed by colocalization analysis *via* Coloc to assess cross-trait consistency of genetic effects. This approach ultimately identified 10 putative shared causal variants across trait pairs (Supplementary Data 7). Furthermore, analysis with HyPrColoc revealed two additional causal variants jointly associated with IgAN and multiple traits (*p* < 0.05; Figure 3(A,B)).

Genetic localization analysis revealed significant enrichment of shared SNVs at loci associated with lipid metabolism pathways and blood pressure regulation (Supplementary Data 7). Notable examples include the *HLA-B* gene, which is located near 15 SNVs shared across 9 trait combinations involving IgAN, and the *HLA-DRA* gene, where 5 SNVs are associated with 5 trait combinations. It is particularly noteworthy that immune-regulatory gene loci, such as *CCDC97*, *PRSS16*, *HLA-B*, and *TRIM26*, harbor pleiotropic SNVs. These findings not only support the hypothesis of immune dysregulation in the pathogenesis of IgAN, but also corroborate previous studies linking these genes to various PVDs [45–49].

### 3.4. Shared genes and pathways

Merely annotating GWAS variants by nearby genes may not fully capture the complexity of pleiotropy. Therefore, we employed four independent methods – TWAS-Fusion, SMR, MAGMA, and GCTA-fastBAT – to infer shared genes. We defined disease-associated genes as those identified by all four methods, ultimately identifying 60 genes (Supplementary Data 8). Notably, *SLC17A1* was shared across IgAN, HTN, and DBP; *FGF5* across IgAN, HTN, and SBP; and *CLEC18C* and *PLEKHO1* were common to IgAN, HTN, and TG.

**Figure 4.**
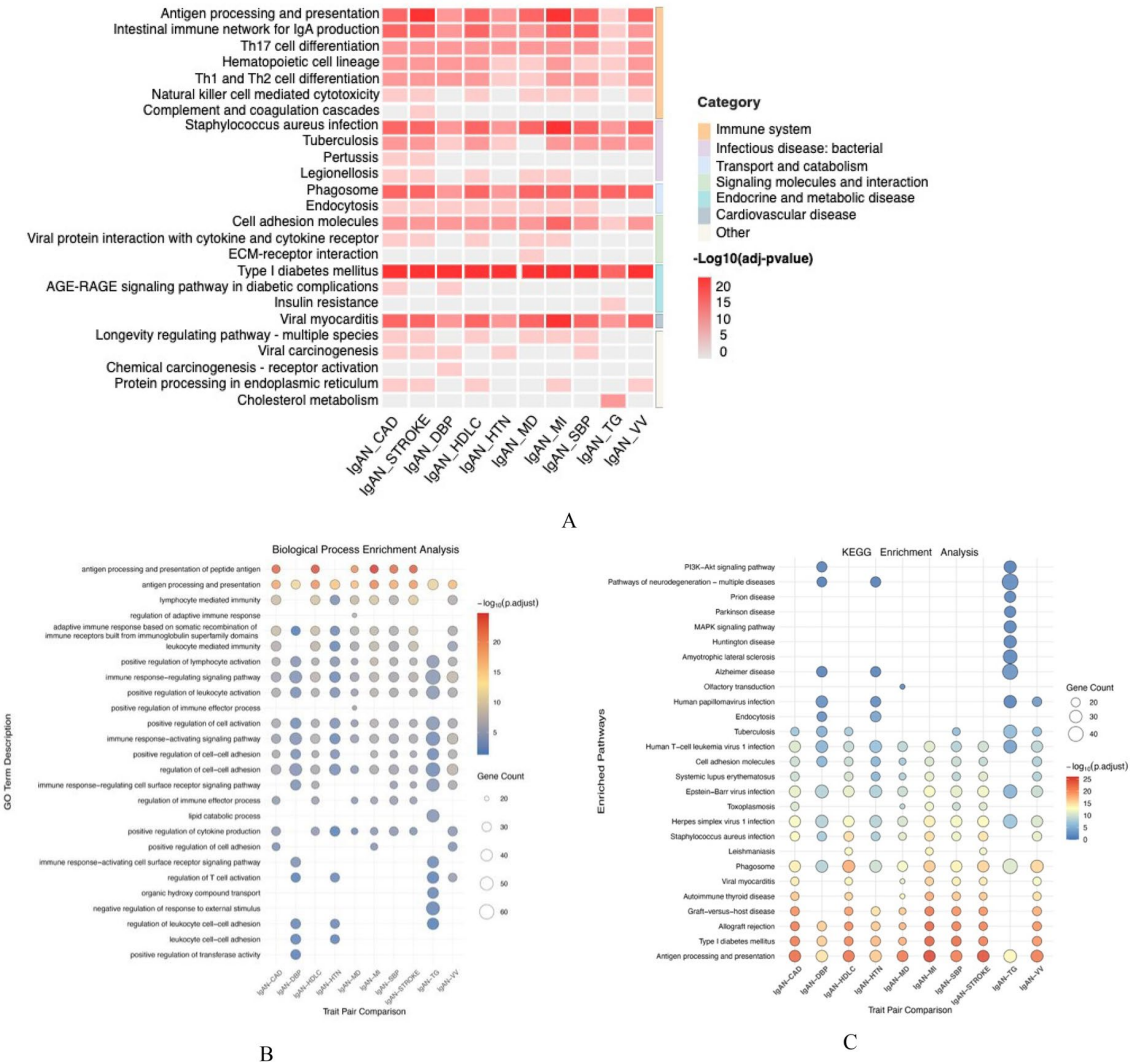
Shared gene enrichment pathways. *Note:* A: KEGG pathway enrichment analysis of shared genes between IgAN and genetically correlated traits. Pathways were grouped by biological mechanisms, and only the top 10 enriched pathways (hypergeometric test, *P* < 0.05) for each trait pair were included. B: Gene Ontology (GO) enrichment analysis of the same shared gene sets. C: KEGG pathway enrichment results based on genes derived from the combined output of four gene-level analysis methods: TWAS-Fusion, SMR, MAGMA, and GCTA-fastBAT. Only the top 10 pathways with FDR-adjusted *P* < 0.05 were shown for each trait pair.

**Figure 5.**
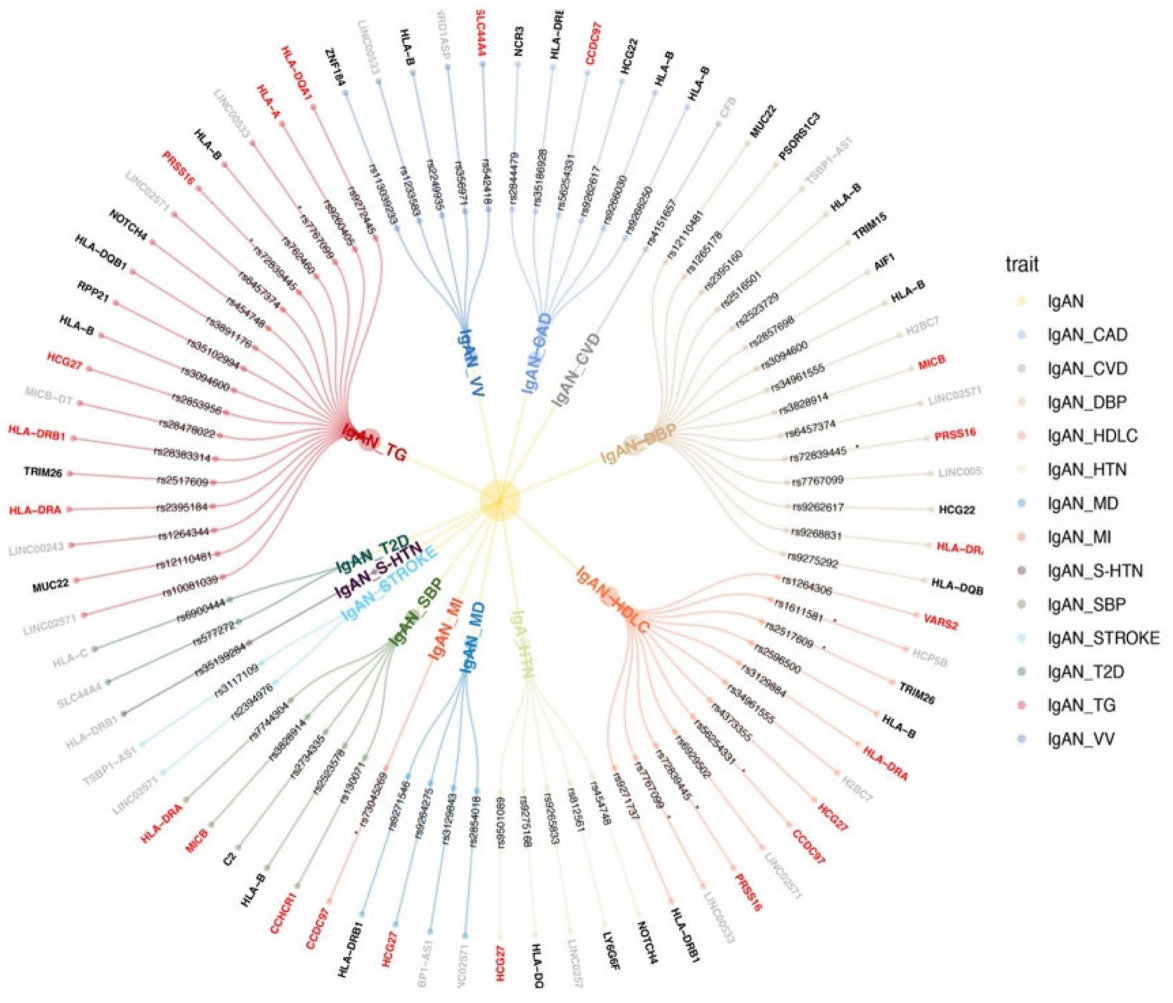
Comorbidity network of IgAN, PVDs, and metabolic characteristics. **Note:** The inner circle displays the SNVs shared between the IgAN characteristic pairs, with 56 shared causal variants (H4 posterior probability PP.H4 > 0.6) marked by an asterisk. The outer circle shows the shared variant genes inferred by Annovar. Genes are highlighted by color to indicate overlap with four gene identification methods: GCTA-fastBAT, MAGMA, TWAS, and SMR. Gray indicates genes not identified by any method, black indicates genes identified by at least one method, and red indicates genes identified by three or more methods.

Moreover, by integrating the genes identified through these four approaches, we provided an overview of the implicated biological pathways. Classified by pathogenic mechanisms, we found these genes to be enriched in pathways related to the immune system, infection, transport and metabolism, as well as cardiovascular diseases (Figure 4(A)), underscoring the dominant role of immune processes. Intriguingly, the IgAN-TG trait pair exhibited the largest number of genes across all ten traits (Figure 4(B,C)). Furthermore, genes associated with lipid metabolism, particularly cholesterol metabolism, were notably distributed within the IgAN-TG trait, further highlighting the potential involvement of lipid metabolism. Additionally, by consolidating the shared SNVs and genes, we constructed a comorbidity network for IgAN, detailing the shared variants and genes between each trait pair (Figure 5).

### 3.5. Tissue- and cell-type specificity

**Figure 6.**
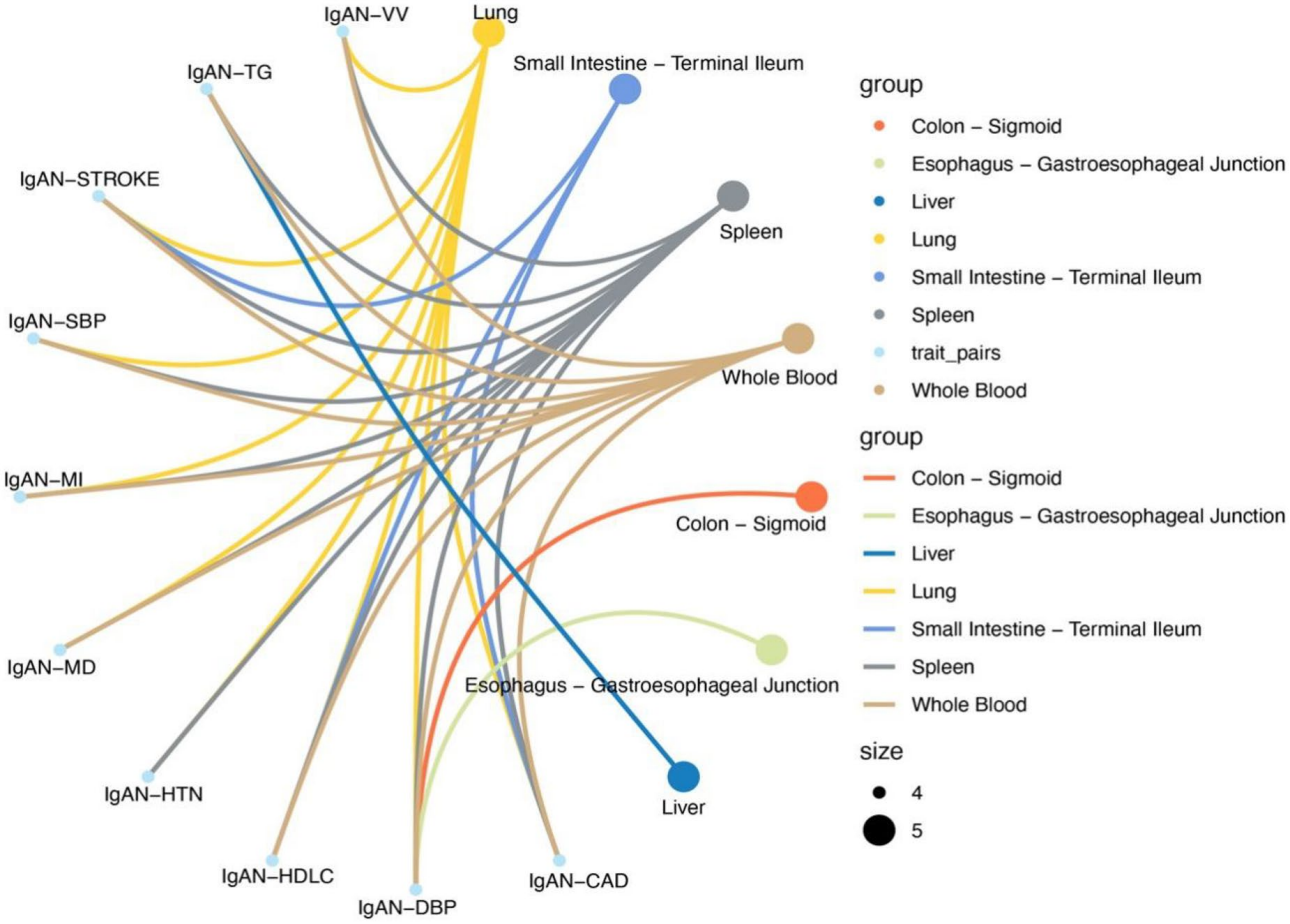
Tissue-specific expression analysis.

**Figure 7.**
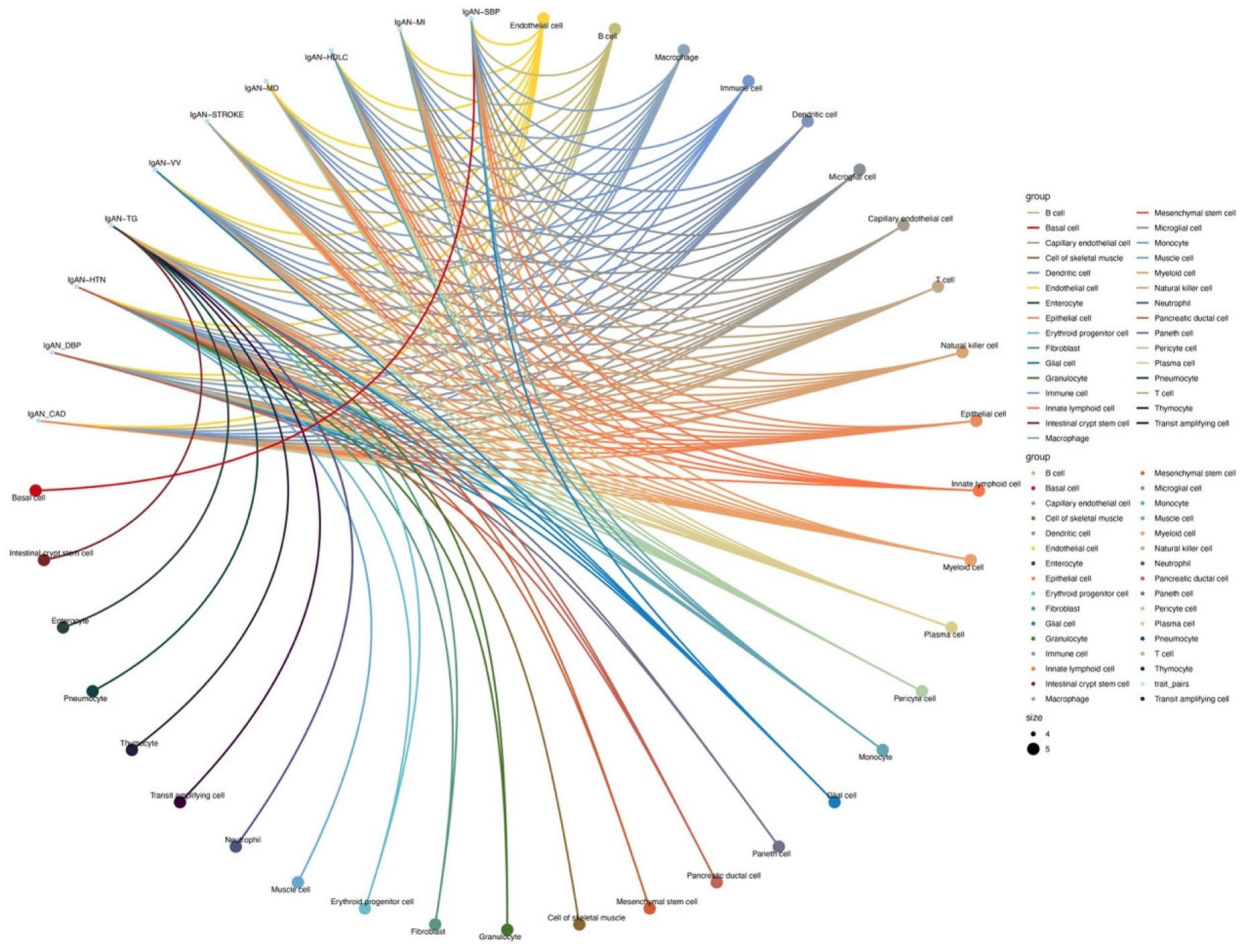
Cell type-specific enrichment analysis.

Shared genes may exert tissue- and cell-specific functions in particular contexts. Integrating the results from two independent methods, this study identified significant enrichment in the spleen, lungs, and peripheral blood (Figure 6), as well as in immune cells, tissue endothelial cells, vascular endothelial cells, epithelial cells, fibroblasts, macrophages, and myeloid cells (Figure 7) across multiple IgAN and trait pairs. These findings suggest that these tissues and cell types are critical hubs for PVDs, immune functions, and metabolic pathways.

Moreover, several specific sharing phenomena were observed. For instance, the liver was uniquely enriched in IgAN and TG, whereas the Colon-Sigmoid and esophagus-gastroesophageal junction were specifically enriched in IgAN and DBP. Interestingly, vascular endothelial cells and immune cells exhibited widespread sharing across various traits, while macrophages, hepatocytes, and endothelial cells from multiple tissues were also associated with IgAN and lipid-related traits. Collectively, these results align with the gene and pathway enrichment analyses, further emphasizing the pivotal roles of lipid metabolism and immune processes in IgAN.

### 3.6. Drug repurposing for IgAN with comorbidities

In this study, disease-associated genes were systematically inferred using four complementary analytical approaches: TWAS-Fusion, SMR, MAGMA, and GCTA-fastBAT. Genes identified concurrently by all four methods were defined as high-confidence disease genes. Consequently, a total of 60 genes were identified as being associated with IgAN, PVDs, and metabolic trait pairs. Given the high comorbidity between PVDs and metabolic disorders, we constructed a drug screening framework based on these shared genes, aiming to identify therapeutic strategies for IgAN patients with comorbid PVDs and metabolic characteristics. Specifically, we employed the pathway pairing scoring method developed by Zhang et al. [38] to match drugs with diseases. Initially, we analyzed the pathological pathways of each trait pair through shared genes, while concurrently integrating authoritative databases such as DrugCentral [41], DGIdb [42], and PharmGKB [43] to construct pharmacological pathway signatures based on drug target genes.

The drug selection process in this study adhered to a targeted approach, focusing primarily on PVD-related targets corresponding to the 60 core genes. After a thorough evaluation, we ultimately identified 40 candidate drugs, which were categorized into seven functional groups: immunomodulators (7 agents, including corticosteroids), antihypertensive drugs (11 agents, such as ACE inhibitors), lipid-lowering agents (8 agents, including fibrates), antidiabetic drugs (3 agents, such as GLP-1 receptor agonists), antiarrhythmic drugs (1 agent), antithrombotic drugs (4 agents, including antiplatelet agents), and antioxidants (6 agents).

**Figure 8.**
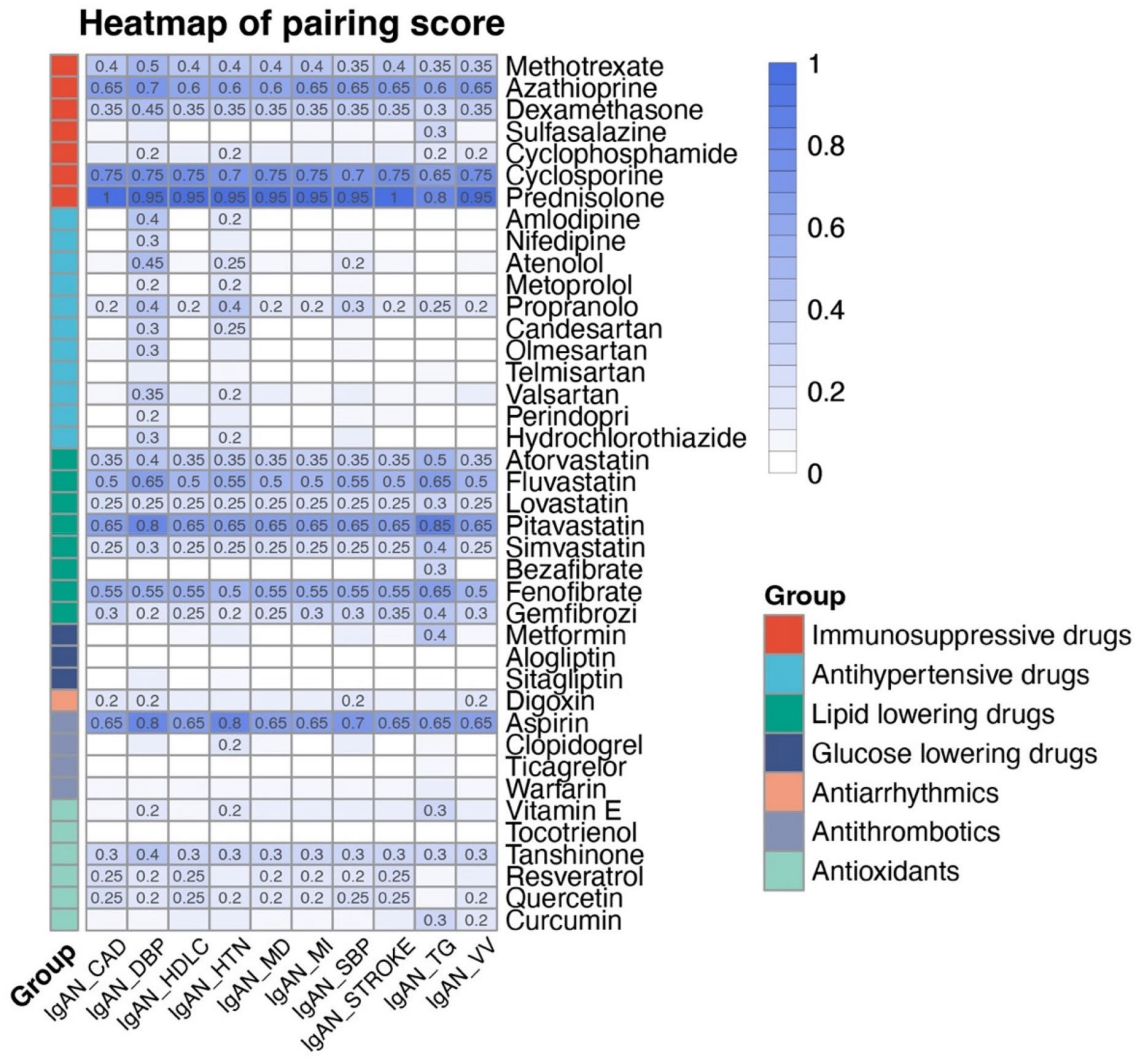
Gene-drug matching scores.

Based on the shared gene–drug pairing score system (with a pairing score threshold of ≥ 0.5; Figure 8), three categories of pharmacological agents with significant therapeutic potential were identified. First, immunomodulatory drugs – predominantly prednisone and cyclosporine – demonstrated notable relevance, with prednisone exhibiting a matching score exceeding 0.8 across all trait pairs. Second, lipid-lowering agents such as pitavastatin and fenofibrate showed high compatibility in the treatment of IgAN comorbid with PVDs, effectively covering a broad spectrum of related phenotypes including HTN, CAD, MI, stroke, and VV. Lastly, antithrombotic agents were also highlighted; notably, aspirin, a classic antiplatelet agent, achieved high scores due to its anti-inflammatory properties and capacity to improve microcirculation, particularly in IgAN patients with concomitant metabolic disturbances such as abnormal DBP, SBP, HDL-C and TG.

## 4. Discussion

In this study, a multidimensional integrative genetic analysis strategy was employed to systematically elucidate the significant comorbid genetic underpinnings between IgAN and a spectrum of PVDs. Subsequent analyses revealed that these disease phenotypes share multiple pleiotropic genetic variants, associated genes, and biological pathways. Moreover, these shared genetic determinants exhibited enrichment or cell-/tissue-specific expression patterns in distinct cellular and anatomical contexts. Collectively, the findings provide a robust theoretical foundation and novel insights into the potential common genetic etiology underlying IgAN and a broad range of vascular and metabolically related disorders.

Based on a large-scale integrative genome-wide analysis, this study identified significant genetic correlations between IgAN and 21 types of PVDs, along with their associated metabolic traits, with LDSC estimates ranging from *r*_g_ = 0.16 to 0.44. Notably, the strongest correlations were observed for CVD (*r*_g_ = 0.41) and HTN (*r*_g_ = 0.33), a trend further validated by GNOVA analysis (*p* < 0.001). Consistent with these genetic findings, epidemiological studies have demonstrated that in patients with IgAN, elevated proteinuria (≥1 g/day) and progression to renal failure are significantly associated with increased risks of cardiovascular events and all-cause mortality [50]. Furthermore, extended SUPERGNOVA analysis revealed that CAD exhibited the greatest number of genetically correlated regions with IgAN, comprising five genome-wide significant loci and 77 suggestive loci. CVD followed closely, with three significant and 73 suggestive loci. Importantly, the highest number of shared SNVs was observed between IgAN and CAD (*N* = 6). These shared genes demonstrated cross-tissue enrichment, particularly in peripheral blood, spleen, and vascular endothelial cells. Collectively, these findings suggest that IgAN is significantly genetically associated with various PVDs, with shared genetic variants enriched in specific tissues, providing important insights into the genetic mechanisms underlying their comorbidity.

The updated Kidney Disease: Improving Global Outcomes (KDIGO) guidelines emphasize that optimized supportive care – including lifestyle modifications such as increased physical activity, weight management, smoking cessation, sodium intake restriction, and cardiovascular risk reduction – forms the cornerstone of IgAN management [51]. In the present study, we comprehensively examined the multidimensional associations between IgAN and its modifiable risk factors (e.g. hypercholesterolemia, obesity, hypertension, and smoking), as well as related metabolic traits, including lipid profiles, blood pressure, and glycemic indicators. LDSC analysis revealed that among all metabolic traits, risk factor–associated phenotypes demonstrated the strongest genetic correlations with IgAN (*r*_g_ = 0.22–0.44), with FHC (*r*_g_ = 0.44), HTN (*r*_g_ = 0.33), and S-HTN (*r*_g_ = 0.33) exhibiting the most pronounced associations. Notably, multiple colocalization analyses identified shared causal variants between IgAN and both DBP and TG. Importantly, shared genetic components were significantly enriched in the spleen, peripheral blood, hepatic dendritic cells, circulating macrophages, and adipose endothelial cells, underscoring the immunometabolic interface of disease. Among all metabolic traits, the pathway enrichment analysis revealed that the genes shared between IgAN and TG-related traits accounted for the largest number of gene counts. In line with this, drug repurposing analysis identified lipid-lowering agents as compelling therapeutic candidates for IgAN patients with coexisting PVDs and metabolic disturbances. Collectively, these findings reinforce the concept that lipid dysfunction may represent a core susceptibility trait underlying the comorbidity of IgAN with PVDs and metabolic abnormalities. Moreover, our data support a robust genetic correlation between blood pressure traits and IgAN. Given that antihypertensive therapy remains one of the most effective non-immunosuppressive strategies in IgAN, its dual benefits are particularly noteworthy: on one hand, it reduces proteinuria, and on the other, it slows the progression of serum creatinine elevation and significantly lowers the risk of ESKD or mortality [52,53]. These observations suggest that the lipid–blood pressure metabolic axis may influence the course of IgAN through shared biological pathways. In contrast, the relationship between glucose metabolism and IgAN appears to be more complex. Although type 2 diabetes mellitus (T2DM), as a known risk factor, showed a significant genetic correlation with IgAN (*r*_g_ = 0.22), individual glycemic traits neither demonstrated significant genetic correlation nor causal associations with IgAN. This discrepancy suggests that dysglycemia may contribute to IgAN progression through indirect or secondary mechanisms.

According to the KDIGO guidelines: In patients with IgAN, if significant proteinuria (≥1 g/d) persists after 3–6 months of standard supportive therapy, resulting in a continuous increase in the risk of renal failure, the current guidelines recommend the prompt initiation of immunosuppressive therapy [51]. Our comprehensive genome-wide enrichment analysis highlights the central role of immune signaling in disease progression: immune-related pathways predominate during disease progression, with several high-frequency shared genes, such as *HLA-B*, *HLA-DRA*, *CCDC97*, *PRSS16*, and *TRIM26*, all of which are closely associated with immune regulation. Specifically, major histocompatibility complex (MHC) genes, including *HLA-B* and *HLA-DRA*, regulate the antigen presentation process, thus driving downstream inflammatory cascades and playing a pivotal role in a variety of immune-mediated diseases[54–57]. In contrast, *CCDC97*, *PRSS16*, and *TRIM26* may participate in the progression of IgAN by modulating immune cell function and maintaining protein homeostasis[58–60]. Furthermore, previous studies have substantiated the close association between MHC polymorphisms and both susceptibility to IgAN and its prognosis. In North American and European populations, *HLA-DR15*, *HLA-DQ5*, and *HLA-DQ6* have been linked to an increased risk of transplant failure, whereas *HLA-DR15* and *HLA-DQ6* confer protective effects on transplant survival [61]. In a Korean cohort, specific *HLA-DRB1*/*DQB1* alleles have been significantly associated with the progression of IgAN to ESKD [62]. Moreover, *HLA-B* has been incorporated into personalized medication strategies; for instance, HLA-B*58:01 has been identified as a predictor of severe allopurinol-induced cutaneous adverse reactions [63]. Although pharmacogenomic research on IgAN remains limited, SNP rs17209237 in the *NR3C1* gene (glucocorticoid receptor) has been significantly correlated with improvements in proteinuria (*p* = 0.021), suggesting that genotype may influence glucocorticoid efficacy and renal function improvement [64]. Transplantation studies also indicate that a high degree of *HLA-DRB1* mismatch results in a reduction in 5-year transplant survival from 94% to 73% (*p* < 0.01) [65], indirectly suggesting that *HLA-DR* polymorphisms may determine the response to immunosuppressive therapy. In conclusion, the integration of immune genetic markers can enhance risk stratification and therapeutic prediction in IgAN, thus advancing personalized and safe treatment strategies. Notably, the immune-regulatory genes *PRSS16* and *TRIM26*, identified in this study, have not yet been fully explored in the context of IgAN, and may serve as promising targets for new drug development and drug repurposing in the future.

In recent years, several novel immunosuppressive therapies have emerged beyond conventional systemic corticosteroids. Notably, the phase III clinical trial NefIgArd (NCT03643965) demonstrated that targeted-release formulation of budesonide (TRF-B) significantly reduced proteinuria and attenuated eGFR decline in patients with IgAN, supporting its therapeutic potential by selectively reducing pathogenic forms of IgA and IgA-containing immune complexes [66]. Similarly, iptacopan (NCT04578834), an alternative complement pathway inhibitor, showed a clinically meaningful reduction in proteinuria, further underscoring the central role of immune dysregulation in IgAN pathogenesis [67]. Moreover, our study shows that the pharmacological-pathological network model constructed based on shared genetic signatures indicates that glucocorticoids exhibit a high degree of concordance across multiple disease phenotypic dimensions, which closely aligns with the immunosuppressive treatment strategies recommended by the KDIGO guidelines [51]. Intriguingly, several immunomodulatory agents currently employed in IgAN – most notably hydroxychloroquine – exert a dual regulatory effect: the drug not only attenuates immune activation *via* Toll‑like receptor inhibition but also significantly improves the lipid profile (total cholesterol/high‑density lipoprotein cholesterol ratio). Indeed, a reduction in the TC/HDL‑C ratio (HR = 2.314; 95% CI = 1.234–4.340; *p* = 0.009) constitutes an independent predictor of ≥ 50 % proteinuria reduction in hydroxychloroquine‑treated patients [68], thereby underscoring the intertwined roles of immunity and lipid metabolism in IgAN pathogenesis. In recent years, renal lipid‑metabolic dysregulation has been increasingly recognized as a pivotal driver of kidney disease progression [8,69]. Retrospective clinical studies have shown that hypertriglyceridemia and hyperuricemia are risk factors for IgAN progression and are more prevalent among patients with an aggressive disease course [12,68,70]. Accumulating evidence further implicates dyslipidemia in the advancement of glomerulonephritis, suggesting shared pathophysiological mechanisms between glomerulosclerosis and atherosclerosis  [71–73]. Consistently, animal studies have demonstrated that lipid‑lowering therapies ameliorate glomerular injury [71,74]. Our data additionally reveal a genetic correlation between IgAN and HDL‑C levels, while drug–gene matching analysis positions lipid‑lowering agents second only to immunosuppressants, thereby highlighting the therapeutic potential of targeting lipid metabolism. This translational framework, founded on high‑frequency shared genes, also points to novel strategies for comorbidity‑oriented interventions in IgAN. For example, several candidate drugs already in widespread clinical use – including renin‑angiotensin system inhibitors (RASi), statins, and the glucocorticoid prednisone – exhibit multifaceted benefits in both IgAN and CVDs. A randomized, placebo‑controlled trial (*n* = 21) reported that six‑month fluvastatin therapy reduced 24‑h proteinuria in mild‑to‑moderate IgAN from a baseline of 837–1100 mg to 494 mg (a 40–55 % decline; *p *< 0.01), thereby illustrating the renoprotective capacity of statins  [75]. Consequently, integrating lipid‑modifying interventions with immune modulation may represent a promising therapeutic avenue for IgAN, particularly in light of the immune‑metabolic cross‑talk elucidated herein. Notably, several lipid‑metabolism susceptibility genes also mediate immune responses. Beyond lipid regulation, statins possess anti‑inflammatory and immunomodulatory properties [76], further accentuating their potential utility in IgAN and its attendant metabolic or immunological comorbidities.

However, this study systematically characterized the risk stratification features of IgAN patients with comorbid conditions, confirming the associations between IgAN and multiple risk factors as well as known biomarkers, and revealing key dimensions of its underlying genetic architecture. Based on these findings, future clinical validation of the identified candidate drugs is warranted. Moreover, integrating patients’ genetic backgrounds and clinical phenotypes will facilitate the development of personalized therapeutic strategies and promote the translational application of precision medicine in IgAN. Importantly, potential differences in drug applicability and safety profiles between adult and pediatric IgAN populations should also be carefully considered [77]. Immunosuppressive agents such as prednisolone and cyclosporine are more commonly used in pediatric IgAN under strict clinical monitoring, whereas lipid-lowering therapies and antiplatelet agents are primarily considered in adult patients with metabolic or vascular comorbidities. Therefore, future large-scale age-stratified GWAS and pharmacogenomic investigations will be essential to refine precision therapeutic strategies across different age groups. Taken together, the present results offer valuable direction for the design of future precision intervention trials grounded in genetic and phenotypic risk markers, particularly in the context of high-risk IgAN subpopulations. As the field of precision nephrology continues to evolve, the integration of genomic data with real-world clinical outcomes will be essential for bridging the gap between research findings and clinical implementation. It should be noted, however, that several high-scoring candidate drugs identified through our bioinformatics pipeline have not yet undergone systematic clinical validation in IgAN cohorts. Consequently, conducting preclinical investigations and prospective clinical trials to evaluate their efficacy and safety in IgAN and its associated comorbidities constitutes a critical next step and a promising opportunity for therapeutic innovation.

This study has several limitations that warrant consideration. First, the IgAN GWAS summary statistics utilized in the present study incorporated both European populations (477,784 individuals, primarily derived from Finnish and UK cohorts) and a large East Asian cohort (175,359 individuals, predominantly of Japanese ancestry). However, the majority of the integrated GWAS datasets, as well as the LD reference structure used for the FM-summary analyses, were still predominantly based on European ancestry populations. Therefore, although individuals of European ancestry constituted the major component of the IgAN GWAS dataset, ancestry-related LD heterogeneity may nevertheless have influenced the fine-mapping results and genetic effect estimates to some extent. Furthermore, despite the inclusion of two major ancestral populations, the genetic background within each ancestry group remained relatively homogeneous, which may limit the generalizability of the identified shared susceptibility loci and molecular mechanisms to other underrepresented populations. Given the substantial differences in allele frequencies and LD patterns across ancestries, the genetic associations identified in the present study should be interpreted cautiously when extrapolated to diverse ethnic groups. Moreover, owing to the lack of age-stratified information in currently publicly available GWAS summary statistics, reliable age-specific genetic datasets for IgAN and related cardiometabolic traits were unavailable. Consequently, robust subgroup analyses according to age could not be performed in the present study, potentially limiting the evaluation of age-dependent genetic effects and disease heterogeneity. Accordingly, future studies incorporating ancestry-specific LD reference panels, larger multi-ethnic cohorts, trans-ethnic fine-mapping strategies, and age-stratified genomic analyses will be essential to further validate the shared genetic architecture across populations and demographic subgroups. Second, although this study focused primarily on the analysis of genetic susceptibility and explored the genetic underpinnings of risk factors such as smoking, it did not incorporate detailed environmental exposure data (e.g. smoking history, levels of air pollution exposure, dietary composition) or individual-level epigenetic regulatory information (e.g. DNA methylation, histone modifications, non-coding RNAs). These non-genetic factors and their complex interactions with the genetic background are likely to play significant roles in the pathogenesis and modulation of IgAN, and therefore merit further investigation. Third, while our study encompassed a broad spectrum of PVDs and metabolic traits, the selection and definition of phenotypes may still present inherent limitations. Emerging or potentially pathophysiologically relevant comorbid features or intermediate phenotypes – such as gut microbiota dysbiosis, specific inflammatory cytokine profiles, or oxidative stress markers – were not systematically incorporated into the current analytical framework. Future studies should consider integrating these multidimensional datasets to achieve a more comprehensive and in-depth understanding of the genetic and multi-omic architecture underlying IgAN and its complex comorbidities. Finally, the inference of candidate gene functions, pathway enrichment, and drug targets in this study relied heavily on annotations from existing public bioinformatics databases. However, such databases may contain incomplete, biased, or outdated information, potentially affecting the accuracy and reliability of certain inferences. Therefore, all associations and mechanistic hypotheses proposed in this study – particularly those involving newly identified shared genes and pathways – require validation through well-designed follow-up functional experiments. These should include gene editing, overexpression or knockdown studies in appropriate cellular and animal models, as well as validation using patient-derived biospecimens.

## 5. Conclusion

This study systematically delineates the complex genetic relationships between IgAN and multiple PVDs as well as related metabolic traits, with a particular emphasis on the significant genetic correlations with cardiometabolic diseases and dyslipidemia. By integrating GWAS data and applying causal inference approaches, we identified a shared genetic architecture between IgAN and key risk factors such as blood pressure and lipid profiles. These findings provide robust evidence supporting the central role of lipid metabolism dysregulation in the development and progression of vascular complications associated with IgAN.

Moreover, cross-trait integrative analyses revealed several shared risk genes and biological pathways, thereby strengthening the genetic basis for immune dysregulation and lipid metabolic disturbances as pivotal mechanisms driving the pathogenesis of IgAN and its comorbidities. Based on these insights, the subsequent drug target exploration suggests that lipid-lowering agents – including statins – may hold promise as potential therapeutic interventions for IgAN-related comorbid conditions, particularly PVDs and metabolic abnormalities. This highlights a novel and clinically relevant direction for future research focusing on lipid metabolic disorders in patients with IgAN.

In summary, our findings not only advance the current understanding of the genetic mechanisms underlying IgAN and its metabolic comorbidities but also lay a critical foundation for the development of more precise risk assessment tools and innovative therapeutic strategies targeting shared pathogenic pathways.

## Supplementary Material

Supplemental Material

## Data Availability

Data are publicly available. The authors confirm that the data supporting the findings of this study are available within the article and its supplementary materials. Supplementary datasets have also been deposited in the Figshare repository and are accessible at: https://figshare.com/s/89b4fe031e4bc27eb800.
